# Synthesis, characterization, and crystal structure of aqua­bis­(4,4′-dimeth­oxy-2,2′-bi­pyridine)[μ-(2*R*,3*R*)-tartrato(4−)]dicopper(II) octa­hydrate

**DOI:** 10.1107/S2056989019008053

**Published:** 2019-06-11

**Authors:** Dennis Wiedemann, Roman-David Kulko, Andreas Grohmann

**Affiliations:** a Institut für Chemie, Technische Universität Berlin, Strasse des 17. Juni 135, 10623 Berlin, Germany

**Keywords:** crystal structure, coordination compound, copper(II) complex, dinuclear complex, bi­pyridine derivative, tartrates, electroless copper baths, hydrogen bonding, π stacks

## Abstract

Typical electroless copper baths (ECBs), which are used to chemically deposit copper on printed circuit boards, consist of an aqueous alkali hydroxide solution, a copper(II) salt, formaldehyde as reducing agent, an l-(+)-tartrate as complexing agent, and a 2,2′-bi­pyridine derivative as stabilizer. Actual speciation and reactivity are, however, largely unknown. Herein, we report on the synthesis and crystal structure of aqua-1κ*O*-bis­(4,4′-dimeth­oxy-2,2′-bi­pyri­dine)-1κ^2^ *N*,*N′*;2κ^2^ *N*,*N′*-[μ-(2*R*,3*R*)-2,3-dioxidosuccinato-1κ^2^ *O* ^1^,*O* ^2^:2κ^2^ *O* ^3^,*O* ^4^]dicopper(II) octa­hydrate, [Cu_2_(C_12_H_12_N_2_O_2_)_2_(C_4_H_2_O_6_)(H_2_O)]·8H_2_O, from an ECB mock-up. The title compound crystallizes in the Sohncke group *P*2_1_ with one chiral dinuclear complex and eight mol­ecules of hydrate water in the asymmetric unit. The expected retention of the tartrato ligand’s absolute configuration was confirmed *via* determination of the absolute structure. The complex mol­ecules exhibit an ansa-like structure with two planar, nearly parallel bi­pyridine ligands, each bound to a copper atom that is connected to the other by a bridging tartrato ‘handle’. The complex and water mol­ecules give rise to a layered supra­molecular structure dominated by alternating π stacks and hydrogen bonds. The understanding of structures *ex situ* is a first step on the way to prolonged stability and improved coating behavior of ECBs.

## Abstract 2

The title compound crystallized from the mock-up of a typical electroless copper bath (ECB) as used to deposit copper on printed circuit boards, consisting of a copper(II) salt, soda lye, l-(+)-tartrate as a complexing agent, and 2,2′-bypyridine derivative as a stabilizer. Its layer-like crystal structure is dominated by extensive π stacking and classical hydrogen bonding.

## Chemical context   

The production of printed circuit boards (PCB) starts with electroless copper deposition (ECD) on electrically non-conductive plastics. Copper is deposited from an alkaline solution of a copper(II) salt and a reducing agent (in general, formaldehyde). The reduction of copper(II) ions proceeds only at pH > 10, thus making methane­diolate (deprotonated formaldehyde hydrate) the actual reactant (Van Den Meerakker, 1981 ▸; Jusys & Vaskelis, 1992 ▸). A complexing agent prevents the precipitation of copper(II) hydroxide (*K*_L_ = 0.16 µmol^3^ L^−3^), which would otherwise occur at pH > 5.7. Since the early development of ECD in 1946, l-(+)-tartrate has commonly been used as complexing agent (Narcus, 1947 ▸). Between pH 11 and 13, it forms bis­(tartrato)copper(II), [Cu(C_4_H_2_O_6_)_2_]^6–^, where each tartaric-acid-derived ligand is quadruply deprotonated. This complex is also known from Fehling’s solution (Fehling, 1848 ▸; Hörner & Klüfers, 2016 ▸). Reactant solutions facilitating ECD, so-called electroless copper baths (ECB), are metastable with respect to the precipitation of metallic copper, making additional stabilizers necessary. Over the past 60 years, a plethora of compounds has been used for this purpose (Agens, 1960 ▸; Saubestre, 1972 ▸), affecting not only the lifetime of ECBs but also the rate of ECD and the physical properties of the deposited copper. Amongst the stabilizers, 2,2′-bi­pyridine and its derivatives are especially popular (Oita *et al.*, 1997 ▸).
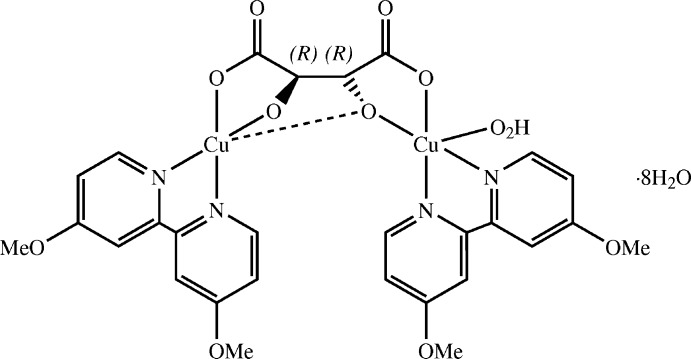


Herein, we report on the crystal structure of a compound that formed from an alkaline solution of a copper(II) salt, a tartrate, and 4,4′-dimeth­oxy-2,2′-bi­pyridine (dmobpy) during the investigation of stabilities of various copper(II) complexes with ligands derived from 2,2′-bi­pyridine (bpy).

## Structural commentary   

**Figure 1:**
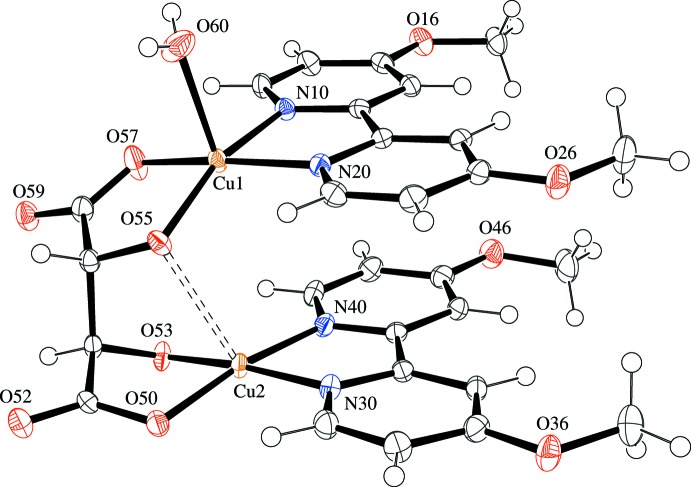
Molecular structure of the title compound as an *ORTEP* plot (complex mol­ecule only, solvent water mol­ecules omitted for clarity). Hydrogen atoms are depicted as spheres with arbitrary radius, all other atoms as displacement ellipsoids of 50% probability. The dashed line indicates a non-bonding short contact.

The compound crystallizes in the Sohncke group *P*2_1_ with one chiral complex mol­ecule and eight mol­ecules of hydration water in the asymmetric unit. The copper(II) ions in the dinuclear complex (see Fig. 1 ▸) are each coordinated by two azine nitro­gen donors, one alcoholate and one carboxyl­ate oxygen donor. The lengths of the respective short bonds (*ca* 1.89–2.00 Å) reflect the formal charge of the donor atoms, while a *cis* configuration is enforced by the structure of the ligand. An additional longer bond to an aqua ligand [*d*(Cu1—O60) = 2.322 (3) Å] augments the coordination environment of Cu1 to a distorted square pyramid. Cu2, on the other hand, is coordinated in a square planar fashion with a short contact to a second alcoholate oxygen atom [*d*(Cu2⋯O55) = 2.549 (2) Å].

The 4,4′-dimeth­oxy-2,2′-bi­pyridine ligands are nearly planar [positional root-mean-square (r.m.s.) deviation excluding hydrogen atoms: 0.032 Å for ligand containing N10 and N20, 0.041 Å for ligand containing N30 and N40], almost parallel [inter­planar angle: 2.70 (4)°], and give rise to intra­molecular π stacks with an average centroid–plane distance of 3.36 (5) Å. Because of this, the overall mol­ecular structure resembles that of ansa compounds, with the tartrato ligand representing the ‘handle’. The tartrato ligand assumes an anti­periplanar (*ap*) conformation with respect to the central bond of the carbon-atom chain. The C—O bonds at the carboxyl­ate donors are synperiplanar (*sp*) to the C—O bonds at the neighboring alcoholate donors.

The absolute structure of the crystal was established *via* anomalous-dispersion effects [the inversion-distinguishing power of the experiment is strong according to Flack & Bernardinelli 2000 ▸)] and matches the absolute configuration of the employed l-(+)-(2*R*,3*R*)-tartrate. The Flack parameter is within the statistical range for an untwinned crystal, thus confirming the enanti­opurity of the complex mol­ecules (Flack & Bernardinelli, 2000 ▸).

## Supra­molecular features   

**Figure 2:**
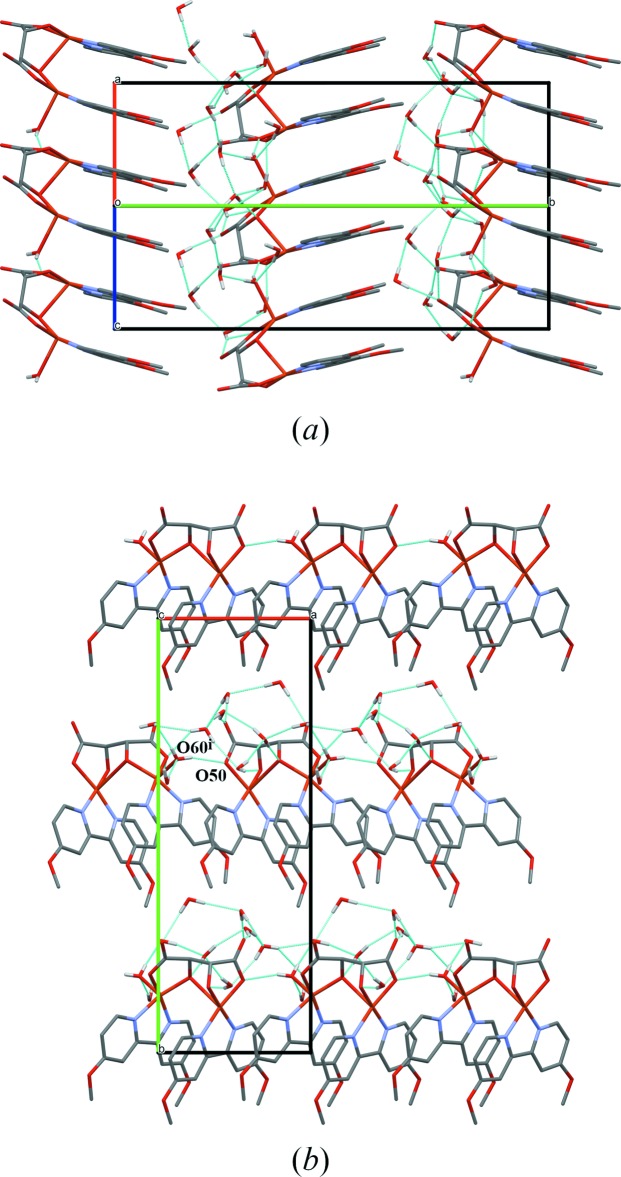
Packing diagram in views along (*a*) the vector *a* + *c* showing the π-stacked bi­pyridine-type ligands and (*b*) the cell vector *c* showing the layer-like arrangement. Dashed bright blue lines indicate hydrogen bonds. Unit-cell boundaries are depicted in black or red/green/blue marking the directions *a*/*b*/*c*, respectively. Symmetry code: (i) *x* − 1, *y*, *z*.

Roughly parallel to {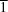
11}, complexes form infinite π stacks, in which the inter­molecular distance of 3.37 (6) Å (average centroid–plane distance) equals the intra­molecular one (see Fig. 2 ▸ *a*). A hydrogen bond from the aqua ligand to the carboxyl­ato oxygen atom O50 of the neighboring mol­ecule in the stack connects the tartrato(4−) ligands, forming an infinite hydrophilic backbone along the *a* direction.

The eight unique water mol­ecules constitute a local network of hydrogen bonds (see Table 1 ▸) in a pocket formed by aqua (donors only) and tartrato ligands (all oxygen atoms as acceptors). The meth­oxy groups do not partake in hydrogen bonding but build a hydro­phobic lining of the pocket. In this way, a front-to-back arrangement of alternating water and complex layers along *b* is formed (see Fig. 2 ▸ *b*).

## Database survey   

The Cambridge Structural Database [CDS 5.40 Update 1 (February 2019); Allen, 2002 ▸; Groom *et al.*, 2016 ▸] contains 33 structures of tartratometal (Co^II^, Cr^III^, Cu^II^, Pd^II^, Pt^II^) complexes with bi­pyridine-related ligands, amongst which twelve contain copper(II). The palladium(II) and platinum(II) complexes are structurally loosely related to [Cu_2_(dmobpy)_2_(μ-C_4_H_2_O_6_)(H_2_O)] in that they form isolated neutral dinuclear complexes [{*M* ^II^ *L*}_2_(μ-C_4_H_2_O_6_)] (*M*: metal, *L*: bi­pyridine-related ligand). Their centers, however, are coordinated in a square-planar fashion without additional longer bonds to oxygen donors.

The copper(II) complexes fall into two groups containing either regular tartrate(2−) or deprotonated tartrate(4−). The former group comprises isolated cationic complexes such as [{Cu(bpy)_2_}_2_(μ-C_4_H_4_O_6_)]^2+^ (Wu *et al.*, 2008 ▸) and poly-/oligomeric complexes such as [Cu(bpy)(μ-C_4_H_4_O_6_)]_*n*_ (Liu *et al.*, 2008 ▸). The latter group, on the other hand, incorporates isolated neutral complexes like aqua-terminated [Cu_2_ *L*_2_(μ-C_4_H_2_O_6_)(H_2_O)] (*L*: bis­[2-pyrid­yl]amine; Li *et al.*, 2006 ▸) or polymeric complexes bridged by carboxyl­ate-*O* donors such as [{Cu(bpy)}_2_(μ_4_-C_4_H_2_O_6_)]_*n*_ presenting Cu_2_O_2_ motifs (Li *et al.*, 2005 ▸).

The closest known relative to the title compound, however, is [Cu_2_(phen)_2_(μ-C_4_H_2_O_6_)(H_2_O)]·8H_2_O (phen: 1,10-phenanthroline), which crystallizes in the same space-group type with comparable cell dimensions (Saha *et al.*, 2011 ▸). Both structures are crystal-chemically homeotypic and differ mainly in the replacement of the 4,4′-meth­oxy groups at the bi­pyridine-like ligands by a 3,3′-(1,2-ethenedi­yl) bridge.

## Synthesis and crystallization   

Copper(II) sulfate penta­hydrate (4.96 g, 19.9 mmol, 1.00 eq), potassium sodium l-(+)-tartrate tetra­hydrate (12.33 g, 43.7 mmol, 2.20 eq), and sodium hydroxide (5.60 g, 140.0 mmol, 7.04 eq) were dissolved in deionized water (1 L), resulting in a solution with pH = 12.8. 4,4′-Dimeth­oxy-2,2′-bi­pyridine (216 mg, 1.00 mmol) was dissolved in sulfuric acid (10 mL, 0.1 mol L^−1^). In a plastic centrifuge tube, the tartratocopper solution (5 mL) was mixed with the bi­pyridine solution (0.12 mmol). The mixture was then filled up to a final volume of 7 mL with deionized water and sodium hydroxide solution to adjust the final pH to 12.8.

After two days of standing unsealed at ambient temperature, dark-blue crystals of [Cu_2_(dmobpy)_2_(μ-C_4_H_2_O_6_)(H_2_O)]·8H_2_O formed.

An infrared (IR) spectrum in attenuated total reflectance (ATR) was acquired from a ground crystal using a Thermo Nicolet iS5 equipped with a Thermo Nicolet iD5 ZnSe sample holder. Bands (*vs*: very strong, *s*: strong, *m*: medium, *w*: weak, *br*: broad) were assigned using literature data (Hesse *et al.*, 1979 ▸; Socrates, 2001 ▸), as well as reference spectra of the dmobpy ligand and potassium sodium l-(+)-tartrate. The crystals were insoluble in common laboratory solvents (alkanes, ethers, alcohols, di­methyl­formamide, dimethyl sulfoxide, and water) at ambient and elevated temperature and decomposed in boiling coordinating solvents. Therefore, we cannot provide data of analyses relying on solutions.

**IR** (ATR): ~*ν* = 3467, 3295 (all *br w*, ν[OH]), 1669 (*s*, ν[OC=O]), 1600 (*vs*, ν[OC—O], ν[C=C], ν[C=N]), 1558 (*vs*, ν[C=C], ν[C=N]), 1499 (*s*), 1476, 1461, 1437, 1418 (all *s*, δ[CH], dmobpy), 1344 (*s*, δ[CH], tartrato), 1317 (*m*), 1280 (*vs*, ν_s_[C—OMe]), 1253 (*s*, tartrato), 1226 (*s*, dmobpy), 1186 (*w*), 1137 (*w*), 1103 (*w*), 1040, 1025, 1016, 1005 (all *s*, ν_as_[C—OMe], ν[C=C], ν[C=N]), 872 (*w*), 851 (*s*, γ[CH]), 838 (*vs*, γ[CH]), 794 (*s*, δ_s_[COO]), 662 (*br m*, ω[COO]), 572 cm^−1^ (*s*, ρ[COO]).

## Refinement   

Crystal data, data collection and structure refinement details are summarized in Table 2 ▸. All non-hydrogen atoms were refined with anisotropic displacement parameters. Hydrogen atoms were located in difference-Fourier maps (for the complex and most water mol­ecules) or their positions were inferred from neighboring sites (for the water mol­ecule containing O68). Carbon-bound hydrogen atoms were refined with standard riding models. Oxygen-bound hydrogen atoms were refined semi-freely with restrained 1,2- [*d*(O—H) ≃ 0.84 (2) Å] and 1,3-distances [*d*(H⋯H) ≃ 1.33 (4) Å], as well as constrained isotropic displacement parameters [*U*_iso_(H) = 1.2*U*_eq_(O)]. Final bond lengths ranged between 0.77 (5) and 0.91 (2) Å with an r.m.s. deviation of 0.036 Å from the target value.

After close inspection of the reflection statistics, data with 2*θ* > 60° (essentially noise) and the high-angle reflection 1 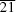 0 (mismeasurement) were excluded from the final refinement. The somewhat lower Friedel pair coverage is due to an inadequate choice of data-collection strategy. Unfortunately, we could not repeat the experiment because of sample loss.

## Supplementary Material

Crystal structure: contains datablock(s) I. DOI: 10.1107/S2056989019008053/zq2247sup1.cif

Structure factors: contains datablock(s) I. DOI: 10.1107/S2056989019008053/zq2247Isup2.hkl

Supporting information file. DOI: 10.1107/S2056989019008053/zq2247Isup3.mol

CCDC reference: 1920829

Additional supporting information:  crystallographic information; 3D view; checkCIF report

## Figures and Tables

**Table 1:** Hydrogen-bond geometry (Å, °)

*D*—H⋯*A*	*D*—H	H⋯*A*	*D*⋯*A*	*D*—H⋯*A*
O60—H60*A*⋯O50^i^	0.81 (5)	2.00 (5)	2.802 (3)	167 (5)
O60—H60*B*⋯O67	0.77 (5)	1.98 (5)	2.750 (4)	177 (5)
O61—H61*A*⋯O65	0.81 (2)	2.02 (3)	2.813 (4)	166 (4)
O61—H61*B*⋯O52^ii^	0.82 (2)	1.92 (2)	2.739 (3)	177 (4)
O62—H62*A*⋯O64	0.79 (2)	2.01 (3)	2.774 (3)	164 (5)
O62—H62*B*⋯O55	0.80 (2)	1.85 (2)	2.650 (3)	174 (5)
O63—H63*A*⋯O59^ii^	0.83 (2)	1.97 (2)	2.806 (3)	178 (4)
O63—H63*B*⋯O61	0.85 (2)	2.13 (2)	2.970 (3)	176 (4)
O64—H64*A*⋯O52	0.81 (2)	2.05 (3)	2.762 (3)	146 (4)
O64—H64*B*⋯O65	0.85 (2)	1.95 (3)	2.719 (3)	149 (4)
O65—H65*A*⋯O59^iii^	0.82 (2)	2.08 (3)	2.871 (3)	162 (4)
O65—H65*B*⋯O66	0.81 (2)	2.01 (2)	2.801 (4)	167 (4)
O66—H66*A*⋯O53^ii^	0.79 (2)	1.91 (3)	2.680 (3)	169 (5)
O66—H66*B*⋯O62	0.80 (2)	2.02 (3)	2.808 (4)	167 (5)
O67—H67*A*⋯O62	0.84 (2)	1.91 (3)	2.737 (4)	168 (5)
O67—H67*B*⋯O59^ii^	0.83 (2)	2.15 (2)	2.973 (3)	178 (5)
O68—H68*A*⋯O57^iii^	0.90 (3)	2.52 (4)	3.236 (4)	138 (5)
O68—H68*B*⋯O60^iv^	0.91 (2)	2.23 (3)	3.129 (5)	169 (5)

Symmetry codes: (i) 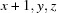; (ii) 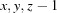; (iii) 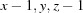; (iv) 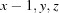.

**Table 2:** Experimental details

Crystal data	
Chemical formula	[Cu_2_(C_12_H_12_N_2_O_2_)_2_(C_4_H_2_O_6_)(H_2_O)]·8H_2_O
*M*_r_	867.75
Crystal system, space group	Monoclinic, *P*2_1_
Temperature (K)	150
*a*, *b*, *c* (Å)	8.5134 (4), 23.7812 (9), 8.9028 (4)
β (°)	101.401 (4)
*V* (Å^3^)	1766.87 (13)
*Z*	2
Radiation type	Mo *K*α
μ (mm^−1^)	1.29
Crystal size (mm)	0.84 × 0.70 × 0.09

Data collection	
Diffractometer	Agilent Xcalibur
Absorption correction	Analytical (*CrysAlis PRO*; Rigaku OD, 2015 ▸)
*T*_min_, *T*_max_	0.440, 0.886
No. of measured, independent and observed [*I* > 2σ(*I*)] reflections	19893, 8971, 8476
*R*_int_	0.021
(sin θ/λ)_max_ (Å^−1^)	0.703

Refinement	
*R*[*F* ^2^ > 2σ(*F* ^2^)], *wR*(*F* ^2^), *S*	0.028, 0.072, 1.04
No. of reflections	8971
No. of parameters	536
No. of restraints	25
H-atom treatment	H-atom parameters constrained for H on C, refined H-atom coordinates only for H on heteroatoms
Δρ_max_, Δρ_min_ (e Å^−3^)	0.49, −0.50
Absolute structure	Flack *x* determined using 2429 quotients [(*I* ^+^)−(*I* ^−^)]/[(*I* ^+^)+(*I* ^−^)] (Parsons *et al.*, 2013 ▸)
Absolute structure parameter	−0.010 (6)

Computer programs: *CrysAlis PRO* (Rigaku OD, 2015 ▸), *SHELXT* (Sheldrick, 2015*a* ▸), *SHELXL* (Sheldrick, 2015*b* ▸), *ORTEP-3 for Windows* (Farrugia, 2012 ▸), *Mercury* (Macrae *et al.*, 2008 ▸), *OLEX2* (Dolomanov *et al.*, 2009 ▸) and *publCIF* (Westrip, 2010 ▸).

## supplementary crystallographic information

### Crystal data

**Table:**

[Cu_2_(C_12_H_12_N_2_O_2_)_2_(C_4_H_2_O_6_)(H_2_O)]·8H_2_O	*F*(000) = 900
*M**_r_* = 867.75	*D*_x_ = 1.631 Mg m^−^^3^
Monoclinic, *P*2_1_	Mo *K*α radiation, λ = 0.71073 Å
*a* = 8.5134 (4) Å	Cell parameters from 8483 reflections
*b* = 23.7812 (9) Å	θ = 3.7–32.5°
*c* = 8.9028 (4) Å	µ = 1.29 mm^−^^1^
β = 101.401 (4)°	*T* = 150 K
*V* = 1766.87 (13) Å^3^	Plate, dark blue
*Z* = 2	0.84 × 0.70 × 0.09 mm

### Data collection

**Table:**

Agilent Xcalibur diffractometer	8971 independent reflections
Radiation source: fine-focus sealed tube, Agilent Enhance	8476 reflections with *I* > 2σ(*I*)
Graphite monochromator	*R*_int_ = 0.021
Detector resolution: 16.3031 pixels mm^-1^	θ_max_ = 30.0°, θ_min_ = 3.5°
ω scans	*h* = −11→11
Absorption correction: analytical (CrysAlis PRO; Rigaku OD, 2015)	*k* = −33→31
*T*_min_ = 0.440, *T*_max_ = 0.886	*l* = −11→12
19893 measured reflections	

### Refinement

**Table:**

Refinement on *F*^2^	Secondary atom site location: difference Fourier map
Least-squares matrix: full	Hydrogen site location: mixed
*R*[*F*^2^ > 2σ(*F*^2^)] = 0.028	Heteroxyz
*wR*(*F*^2^) = 0.072	*w* = 1/[σ^2^(*F*_o_^2^) + (0.0455*P*)^2^ + 0.0799*P*] where *P* = (*F*_o_^2^ + 2*F*_c_^2^)/3
*S* = 1.04	(Δ/σ)_max_ = 0.001
8971 reflections	Δρ_max_ = 0.49 e Å^−^^3^
536 parameters	Δρ_min_ = −0.50 e Å^−^^3^
25 restraints	Absolute structure: Flack *x* determined using 2429 quotients [(*I*^+^)-(*I*^-^)]/[(*I*^+^)+(*I*^-^)] (Parsons *et al.*, 2013)
Primary atom site location: dual	Absolute structure parameter: −0.010 (6)

### Special details

**Table:**

Geometry. All esds (except the esd in the dihedral angle between two l.s. planes) are estimated using the full covariance matrix. The cell esds are taken into account individually in the estimation of esds in distances, angles and torsion angles; correlations between esds in cell parameters are only used when they are defined by crystal symmetry. An approximate (isotropic) treatment of cell esds is used for estimating esds involving l.s. planes. ———————————————————————— l.s. plane 1 ———————————————————————— Equation (*x*, *y*, *z* in crystal coordinates): –6.8237 (16) *x* + 5.2625 (12) *y* + 6.259 (2) *z* = 3.953 (6) Defining atoms and their deviation from the plane: N30: –0.014 (2) Å C31: –0.046 (3) Å C32: –0.024 (3) Å C33: 0.024 (3) Å C34: 0.042 (3) Å C35: 0.024 (2) Å O36: 0.055 (2) Å C37: –0.075 (3) Å N40: 0.042 (2) Å C41: 0.018 (3) Å C42: –0.029 (3) Å C43: –0.033 (3) Å C44: –0.014 (3) Å C45: 0.023 (2) Å O46: –0.059 (2) Å C47: 0.065 (3) Å Rms deviation: 0.041 Å ———————————————————————— l.s. plane 2 ———————————————————————— Equation (*x*, *y*, *z* in crystal coordinates): –6.6296 (17) *x* + 6.166 (11) *y* + 6.3561 (18) *z* = 1.306 (6) Defining atoms and their deviation from the plane: N10: –0.025 (2) Å C11: –0.035 (2) Å C12: –0.025 (3) Å C13: –0.001 (3) Å C14: 0.002 (3) Å C15: –0.014 (2) Å O16: 0.013 (2) Å C17: 0.056 (3) Å N20: 0.074 (2) Å C21: 0.043 (2) Å C22: –0.003 (3) Å C23: –0.019 (3) Å C24: –0.046 (2) Å C25: 0.003 (2) Å O26: 0.004 (2) Å C27: –0.026 (3) Å Rms deviation: 0.032 Å ———————————————————————— Angle between planes 1 and 2: 2.70 (4)°
Refinement. Hydrogen atoms were located on difference Fourier maps for the complex and most water molecules or inferred from neighbouring sites for the water molecule containing O68. Hydrogen positions were refined semi-freely for oxygen-bound atoms with *d*(O—H) ≈ 0.84 (2) Å, *d*(H···H) ≈ 1.33 Å, and *U*_iso_(H) = 1.2*U*_eq_(O).

### Fractional atomic coordinates and isotropic or equivalent isotropic displacement parameters (Å^2^)

**Table:**

	*x*	*y*	*z*	*U*_iso_*/*U*_eq_	
C11	1.2715 (3)	0.41601 (12)	1.1226 (3)	0.0182 (5)	
H11	1.283890	0.379796	1.168495	0.022*	
C12	1.3710 (3)	0.45876 (13)	1.1865 (3)	0.0200 (5)	
H12	1.451278	0.452042	1.275142	0.024*	
C13	1.3535 (3)	0.51201 (13)	1.1204 (3)	0.0170 (5)	
C14	1.2344 (3)	0.52043 (12)	0.9885 (3)	0.0164 (5)	
H14	1.219570	0.556191	0.940163	0.020*	
C15	1.1396 (3)	0.47504 (12)	0.9311 (3)	0.0145 (5)	
C17	1.4390 (4)	0.60720 (13)	1.1263 (4)	0.0245 (6)	
H17A	1.456712	0.606259	1.020808	0.037*	
H17B	1.518812	0.631776	1.188359	0.037*	
H17C	1.331302	0.621650	1.126726	0.037*	
C21	0.8061 (3)	0.42851 (13)	0.6374 (3)	0.0193 (5)	
H21	0.749051	0.394330	0.612752	0.023*	
C22	0.7652 (3)	0.47396 (13)	0.5435 (3)	0.0200 (5)	
H22	0.681591	0.471184	0.455702	0.024*	
C23	0.8477 (3)	0.52418 (13)	0.5782 (3)	0.0178 (5)	
C24	0.9754 (3)	0.52630 (12)	0.7051 (3)	0.0159 (5)	
H24	1.037174	0.559525	0.729475	0.019*	
C25	1.0079 (3)	0.47817 (11)	0.7933 (3)	0.0145 (5)	
C27	0.8802 (4)	0.62092 (14)	0.5171 (4)	0.0300 (7)	
H27A	0.865758	0.635393	0.616547	0.045*	
H27B	0.836620	0.647981	0.436580	0.045*	
H27C	0.994577	0.615298	0.519038	0.045*	
C31	0.3834 (3)	0.43168 (13)	0.6794 (3)	0.0205 (5)	
H31	0.349432	0.393596	0.668076	0.025*	
C32	0.3125 (4)	0.46999 (14)	0.5734 (3)	0.0213 (5)	
H32	0.230184	0.458718	0.490547	0.026*	
C33	0.3622 (3)	0.52578 (13)	0.5883 (3)	0.0193 (5)	
C34	0.4843 (3)	0.54103 (12)	0.7115 (3)	0.0166 (5)	
H34	0.521299	0.578745	0.724106	0.020*	
C35	0.5496 (3)	0.49946 (12)	0.8148 (3)	0.0154 (5)	
C37	0.3456 (4)	0.61790 (14)	0.4770 (4)	0.0290 (7)	
H37A	0.338665	0.637059	0.572958	0.043*	
H37B	0.280905	0.638229	0.390659	0.043*	
H37C	0.457485	0.616927	0.464978	0.043*	
C41	0.8415 (3)	0.46603 (13)	1.1602 (3)	0.0192 (5)	
H41	0.869537	0.433356	1.220971	0.023*	
C42	0.9236 (3)	0.51491 (13)	1.2011 (3)	0.0213 (6)	
H42	1.008567	0.515850	1.288088	0.026*	
C43	0.8816 (3)	0.56337 (13)	1.1140 (3)	0.0204 (5)	
C44	0.7578 (3)	0.56044 (12)	0.9846 (3)	0.0168 (5)	
H44	0.727153	0.592557	0.922194	0.020*	
C45	0.6817 (3)	0.50923 (12)	0.9505 (3)	0.0160 (5)	
C47	0.9181 (4)	0.66188 (15)	1.0865 (5)	0.0335 (7)	
H47A	0.933207	0.659815	0.980339	0.050*	
H47B	0.983627	0.692454	1.139794	0.050*	
H47C	0.804933	0.669051	1.087414	0.050*	
C51	0.5003 (3)	0.28733 (12)	0.9647 (3)	0.0164 (5)	
C54	0.6577 (3)	0.29477 (12)	1.0809 (3)	0.0152 (5)	
H54	0.664279	0.266896	1.165969	0.018*	
C56	0.7939 (3)	0.28407 (12)	0.9911 (3)	0.0148 (5)	
H56	0.776060	0.246864	0.938153	0.018*	
C58	0.9527 (3)	0.28248 (13)	1.1070 (3)	0.0192 (5)	
N10	1.1565 (3)	0.42400 (10)	0.9965 (3)	0.0151 (4)	
N20	0.9238 (3)	0.43016 (10)	0.7633 (3)	0.0150 (4)	
N30	0.4999 (3)	0.44568 (10)	0.7997 (3)	0.0175 (4)	
N40	0.7216 (3)	0.46292 (10)	1.0359 (3)	0.0167 (4)	
O16	1.4539 (3)	0.55171 (10)	1.1887 (2)	0.0223 (4)	
O26	0.7974 (3)	0.56828 (10)	0.4865 (2)	0.0239 (4)	
O36	0.2861 (3)	0.56124 (10)	0.4806 (2)	0.0244 (4)	
O46	0.9656 (3)	0.60980 (10)	1.1623 (3)	0.0265 (5)	
O50	0.4432 (2)	0.33278 (9)	0.8974 (2)	0.0206 (4)	
O52	0.4392 (3)	0.24029 (9)	0.9335 (2)	0.0217 (4)	
O53	0.6716 (3)	0.34935 (8)	1.1395 (2)	0.0185 (4)	
O55	0.7926 (2)	0.32664 (9)	0.8810 (2)	0.0170 (4)	
O57	1.0554 (3)	0.31931 (10)	1.0907 (3)	0.0293 (5)	
O59	0.9761 (3)	0.24641 (10)	1.2100 (2)	0.0233 (4)	
O60	1.1234 (3)	0.32417 (11)	0.7440 (3)	0.0333 (6)	
H60A	1.217 (6)	0.321 (2)	0.788 (5)	0.040*	
H60B	1.084 (6)	0.300 (2)	0.691 (5)	0.040*	
Cu1	0.98891 (3)	0.36813 (2)	0.91447 (4)	0.01545 (7)	
Cu2	0.59542 (4)	0.39447 (2)	0.96609 (4)	0.01590 (7)	
O61	0.4573 (3)	0.17342 (10)	0.1868 (3)	0.0273 (5)	
H61A	0.421 (5)	0.1934 (16)	0.245 (4)	0.033*	
H61B	0.450 (5)	0.1945 (16)	0.112 (3)	0.033*	
O62	0.6964 (3)	0.27767 (12)	0.6111 (3)	0.0283 (5)	
H62A	0.633 (4)	0.2548 (15)	0.623 (5)	0.034*	
H62B	0.720 (5)	0.2935 (17)	0.692 (3)	0.034*	
O63	0.7991 (3)	0.14628 (10)	0.1929 (3)	0.0292 (5)	
H63A	0.852 (4)	0.1759 (14)	0.196 (5)	0.035*	
H63B	0.703 (3)	0.1559 (18)	0.192 (5)	0.035*	
O64	0.4289 (3)	0.21351 (11)	0.6298 (3)	0.0284 (5)	
H64A	0.403 (5)	0.2303 (18)	0.701 (3)	0.034*	
H64B	0.364 (4)	0.2296 (18)	0.558 (3)	0.034*	
O65	0.3108 (3)	0.25427 (12)	0.3445 (3)	0.0295 (5)	
H65A	0.219 (3)	0.2590 (18)	0.297 (4)	0.035*	
H65B	0.359 (5)	0.2835 (13)	0.351 (5)	0.035*	
O66	0.5228 (3)	0.34554 (12)	0.3780 (3)	0.0312 (5)	
H66A	0.562 (5)	0.3509 (19)	0.306 (3)	0.037*	
H66B	0.582 (4)	0.330 (2)	0.446 (4)	0.037*	
O67	0.9808 (3)	0.24228 (14)	0.5446 (3)	0.0396 (6)	
H67A	0.887 (4)	0.251 (2)	0.552 (5)	0.048*	
H67B	0.982 (6)	0.243 (2)	0.452 (3)	0.048*	
O68	0.0583 (5)	0.37893 (15)	0.4180 (4)	0.0594 (9)	
H68A	0.037 (7)	0.3483 (18)	0.359 (5)	0.071*	
H68B	0.065 (7)	0.366 (2)	0.515 (3)	0.071*	

### Atomic displacement parameters (Å^2^)

**Table:**

	*U*^11^	*U*^22^	*U*^33^	*U*^12^	*U*^13^	*U*^23^
C11	0.0168 (12)	0.0151 (13)	0.0211 (12)	−0.0010 (10)	0.0000 (9)	0.0043 (10)
C12	0.0184 (12)	0.0220 (14)	0.0180 (12)	−0.0040 (11)	−0.0004 (10)	0.0012 (11)
C13	0.0165 (11)	0.0191 (13)	0.0158 (11)	−0.0048 (10)	0.0038 (9)	−0.0034 (10)
C14	0.0170 (11)	0.0150 (12)	0.0175 (11)	−0.0018 (10)	0.0042 (9)	0.0004 (10)
C15	0.0121 (11)	0.0158 (13)	0.0157 (11)	−0.0003 (9)	0.0033 (9)	−0.0004 (9)
C17	0.0309 (15)	0.0152 (13)	0.0274 (14)	−0.0069 (11)	0.0058 (12)	−0.0028 (11)
C21	0.0177 (12)	0.0203 (14)	0.0196 (12)	−0.0036 (10)	0.0031 (9)	−0.0013 (11)
C22	0.0165 (12)	0.0229 (14)	0.0189 (12)	−0.0005 (10)	−0.0007 (9)	0.0004 (11)
C23	0.0169 (12)	0.0186 (13)	0.0179 (11)	0.0022 (10)	0.0032 (9)	0.0026 (10)
C24	0.0151 (11)	0.0148 (12)	0.0183 (11)	0.0004 (9)	0.0048 (9)	−0.0007 (10)
C25	0.0140 (11)	0.0145 (12)	0.0157 (10)	0.0014 (9)	0.0046 (9)	−0.0005 (9)
C27	0.0337 (16)	0.0181 (15)	0.0348 (16)	0.0021 (12)	−0.0014 (13)	0.0074 (12)
C31	0.0218 (12)	0.0159 (13)	0.0233 (12)	−0.0016 (10)	0.0030 (10)	−0.0001 (11)
C32	0.0222 (12)	0.0211 (14)	0.0193 (12)	−0.0021 (11)	0.0008 (10)	0.0011 (11)
C33	0.0220 (13)	0.0204 (14)	0.0161 (11)	0.0038 (11)	0.0055 (10)	0.0036 (10)
C34	0.0209 (12)	0.0122 (12)	0.0179 (11)	0.0012 (10)	0.0068 (9)	0.0015 (9)
C35	0.0167 (11)	0.0138 (12)	0.0167 (11)	0.0005 (9)	0.0061 (9)	−0.0003 (10)
C37	0.0421 (18)	0.0213 (16)	0.0224 (14)	0.0019 (13)	0.0037 (13)	0.0062 (12)
C41	0.0242 (13)	0.0146 (13)	0.0189 (12)	0.0047 (10)	0.0043 (10)	0.0012 (10)
C42	0.0209 (13)	0.0214 (15)	0.0200 (12)	0.0027 (11)	0.0007 (10)	−0.0017 (11)
C43	0.0208 (13)	0.0176 (13)	0.0239 (12)	−0.0001 (10)	0.0074 (10)	−0.0036 (11)
C44	0.0189 (12)	0.0129 (12)	0.0195 (11)	0.0005 (10)	0.0057 (9)	−0.0012 (10)
C45	0.0179 (11)	0.0147 (13)	0.0174 (11)	0.0021 (9)	0.0079 (9)	0.0004 (10)
C47	0.0354 (17)	0.0169 (15)	0.0461 (19)	−0.0074 (13)	0.0026 (14)	0.0008 (14)
C51	0.0161 (11)	0.0152 (13)	0.0193 (12)	−0.0019 (10)	0.0067 (9)	0.0033 (10)
C54	0.0177 (11)	0.0101 (12)	0.0176 (11)	−0.0009 (9)	0.0028 (9)	0.0014 (9)
C56	0.0144 (11)	0.0133 (12)	0.0155 (10)	−0.0021 (9)	0.0002 (8)	0.0000 (9)
C58	0.0179 (12)	0.0160 (13)	0.0216 (12)	−0.0012 (10)	−0.0012 (9)	0.0015 (10)
N10	0.0141 (9)	0.0144 (11)	0.0161 (9)	−0.0022 (8)	0.0014 (8)	0.0000 (8)
N20	0.0142 (9)	0.0138 (11)	0.0168 (9)	0.0004 (8)	0.0024 (7)	0.0007 (8)
N30	0.0196 (10)	0.0129 (11)	0.0199 (10)	0.0012 (9)	0.0038 (8)	0.0013 (9)
N40	0.0190 (10)	0.0131 (11)	0.0184 (10)	0.0024 (9)	0.0044 (8)	0.0000 (9)
O16	0.0244 (10)	0.0199 (11)	0.0207 (9)	−0.0093 (8)	−0.0003 (8)	−0.0036 (8)
O26	0.0247 (10)	0.0198 (10)	0.0241 (10)	0.0019 (8)	−0.0026 (8)	0.0062 (8)
O36	0.0289 (11)	0.0200 (11)	0.0218 (9)	0.0034 (9)	−0.0011 (8)	0.0063 (8)
O46	0.0256 (10)	0.0192 (11)	0.0326 (11)	−0.0032 (9)	0.0005 (8)	−0.0027 (9)
O50	0.0162 (9)	0.0165 (10)	0.0281 (10)	−0.0012 (8)	0.0017 (8)	0.0054 (8)
O52	0.0233 (10)	0.0175 (10)	0.0240 (9)	−0.0051 (8)	0.0039 (8)	0.0022 (8)
O53	0.0294 (10)	0.0093 (9)	0.0169 (8)	0.0006 (7)	0.0048 (7)	−0.0002 (7)
O55	0.0145 (8)	0.0188 (10)	0.0166 (8)	−0.0041 (7)	0.0007 (6)	0.0039 (7)
O57	0.0209 (10)	0.0231 (12)	0.0374 (12)	−0.0077 (9)	−0.0102 (9)	0.0142 (10)
O59	0.0221 (10)	0.0192 (10)	0.0262 (10)	−0.0021 (8)	−0.0013 (8)	0.0067 (9)
O60	0.0181 (10)	0.0263 (13)	0.0525 (15)	0.0028 (9)	−0.0001 (10)	−0.0113 (11)
Cu1	0.01300 (13)	0.01261 (15)	0.01936 (14)	−0.00242 (12)	−0.00014 (10)	0.00193 (13)
Cu2	0.01896 (15)	0.01035 (14)	0.01850 (14)	0.00071 (13)	0.00396 (10)	0.00248 (12)
O61	0.0383 (13)	0.0166 (11)	0.0283 (11)	0.0021 (9)	0.0101 (10)	0.0035 (9)
O62	0.0311 (12)	0.0342 (14)	0.0183 (10)	−0.0094 (10)	0.0014 (9)	−0.0032 (9)
O63	0.0298 (11)	0.0163 (11)	0.0406 (13)	−0.0026 (9)	0.0051 (10)	0.0010 (10)
O64	0.0360 (13)	0.0240 (12)	0.0227 (10)	−0.0019 (10)	0.0002 (9)	0.0003 (9)
O65	0.0235 (11)	0.0343 (14)	0.0274 (11)	0.0028 (10)	−0.0032 (9)	0.0015 (10)
O66	0.0395 (13)	0.0320 (13)	0.0245 (11)	0.0059 (11)	0.0121 (9)	0.0053 (10)
O67	0.0366 (14)	0.0479 (17)	0.0336 (13)	0.0007 (13)	0.0052 (11)	−0.0124 (12)
O68	0.085 (2)	0.038 (2)	0.0514 (18)	−0.0002 (17)	0.0039 (17)	0.0004 (14)

### Geometric parameters (Å, º)

**Table:**

C11—H11	0.9500	C42—C43	1.396 (4)
C11—C12	1.373 (4)	C43—C44	1.401 (4)
C11—N10	1.350 (3)	C43—O46	1.340 (4)
C12—H12	0.9500	C44—H44	0.9500
C12—C13	1.392 (4)	C44—C45	1.385 (4)
C13—C14	1.406 (4)	C45—N40	1.344 (4)
C13—O16	1.336 (3)	C47—H47A	0.9800
C14—H14	0.9500	C47—H47B	0.9800
C14—C15	1.384 (4)	C47—H47C	0.9800
C15—C25	1.491 (4)	C47—O46	1.430 (4)
C15—N10	1.342 (4)	C51—C54	1.532 (4)
C17—H17A	0.9800	C51—O50	1.284 (4)
C17—H17B	0.9800	C51—O52	1.242 (4)
C17—H17C	0.9800	C54—H54	1.0000
C17—O16	1.428 (4)	C54—C56	1.554 (4)
C21—H21	0.9500	C54—O53	1.395 (3)
C21—C22	1.368 (4)	C56—H56	1.0000
C21—N20	1.349 (3)	C56—C58	1.530 (4)
C22—H22	0.9500	C56—O55	1.408 (3)
C22—C23	1.389 (4)	C58—O57	1.266 (4)
C23—C24	1.406 (4)	C58—O59	1.242 (4)
C23—O26	1.346 (3)	N10—Cu1	1.980 (2)
C24—H24	0.9500	N20—Cu1	2.000 (2)
C24—C25	1.385 (4)	N30—Cu2	1.965 (2)
C25—N20	1.346 (4)	N40—Cu2	1.981 (2)
C27—H27A	0.9800	O50—Cu2	1.973 (2)
C27—H27B	0.9800	O53—Cu2	1.887 (2)
C27—H27C	0.9800	O55—Cu1	1.9128 (19)
C27—O26	1.436 (4)	O57—Cu1	1.944 (2)
C31—H31	0.9500	O60—H60A	0.81 (5)
C31—C32	1.364 (4)	O60—H60B	0.77 (5)
C31—N30	1.350 (4)	O60—Cu1	2.322 (3)
C32—H32	0.9500	O61—H61A	0.81 (2)
C32—C33	1.391 (4)	O61—H61B	0.82 (2)
C33—C34	1.402 (4)	O62—H62A	0.79 (2)
C33—O36	1.343 (3)	O62—H62B	0.80 (2)
C34—H34	0.9500	O63—H63A	0.83 (2)
C34—C35	1.389 (4)	O63—H63B	0.85 (2)
C35—C45	1.497 (4)	O64—H64A	0.81 (2)
C35—N30	1.345 (4)	O64—H64B	0.85 (2)
C37—H37A	0.9800	O65—H65A	0.82 (2)
C37—H37B	0.9800	O65—H65B	0.81 (2)
C37—H37C	0.9800	O66—H66A	0.79 (2)
C37—O36	1.442 (4)	O66—H66B	0.80 (2)
C41—H41	0.9500	O67—H67A	0.84 (2)
C41—C42	1.368 (4)	O67—H67B	0.83 (2)
C41—N40	1.351 (4)	O68—H68A	0.90 (3)
C42—H42	0.9500	O68—H68B	0.91 (2)
			
O55···Cu2	2.549 (2)		
			
C12—C11—H11	119.1	H47A—C47—H47B	109.5
N10—C11—H11	119.1	H47A—C47—H47C	109.5
N10—C11—C12	121.8 (3)	H47B—C47—H47C	109.5
C11—C12—H12	120.2	O46—C47—H47A	109.5
C11—C12—C13	119.6 (2)	O46—C47—H47B	109.5
C13—C12—H12	120.2	O46—C47—H47C	109.5
C12—C13—C14	118.8 (3)	O50—C51—C54	114.7 (2)
O16—C13—C12	116.4 (2)	O52—C51—C54	121.8 (2)
O16—C13—C14	124.7 (3)	O52—C51—O50	123.4 (2)
C13—C14—H14	121.0	C51—C54—H54	110.2
C15—C14—C13	118.0 (3)	C51—C54—C56	106.0 (2)
C15—C14—H14	121.0	C56—C54—H54	110.2
C14—C15—C25	123.6 (3)	O53—C54—C51	111.0 (2)
N10—C15—C14	122.7 (2)	O53—C54—H54	110.2
N10—C15—C25	113.6 (2)	O53—C54—C56	109.1 (2)
H17A—C17—H17B	109.5	C54—C56—H56	109.1
H17A—C17—H17C	109.5	C58—C56—C54	107.9 (2)
H17B—C17—H17C	109.5	C58—C56—H56	109.1
O16—C17—H17A	109.5	O55—C56—C54	109.6 (2)
O16—C17—H17B	109.5	O55—C56—H56	109.1
O16—C17—H17C	109.5	O55—C56—C58	111.8 (2)
C22—C21—H21	118.6	O57—C58—C56	116.4 (2)
N20—C21—H21	118.6	O59—C58—C56	120.4 (2)
N20—C21—C22	122.8 (3)	O59—C58—O57	123.3 (3)
C21—C22—H22	120.4	C11—N10—Cu1	124.3 (2)
C21—C22—C23	119.2 (2)	C15—N10—C11	119.1 (2)
C23—C22—H22	120.4	C15—N10—Cu1	116.08 (17)
C22—C23—C24	119.1 (3)	C21—N20—Cu1	126.8 (2)
O26—C23—C22	116.7 (2)	C25—N20—C21	117.9 (2)
O26—C23—C24	124.2 (3)	C25—N20—Cu1	115.24 (17)
C23—C24—H24	121.2	C31—N30—Cu2	125.1 (2)
C25—C24—C23	117.5 (3)	C35—N30—C31	118.7 (2)
C25—C24—H24	121.2	C35—N30—Cu2	116.03 (19)
C24—C25—C15	122.8 (2)	C41—N40—Cu2	125.2 (2)
N20—C25—C15	113.9 (2)	C45—N40—C41	119.0 (3)
N20—C25—C24	123.4 (2)	C45—N40—Cu2	115.79 (18)
H27A—C27—H27B	109.5	C13—O16—C17	118.4 (2)
H27A—C27—H27C	109.5	C23—O26—C27	118.6 (2)
H27B—C27—H27C	109.5	C33—O36—C37	118.7 (2)
O26—C27—H27A	109.5	C43—O46—C47	118.6 (2)
O26—C27—H27B	109.5	C51—O50—Cu2	108.45 (17)
O26—C27—H27C	109.5	C54—O53—Cu2	103.46 (15)
C32—C31—H31	118.7	C56—O55—Cu1	112.06 (15)
N30—C31—H31	118.7	C56—O55—Cu2	99.39 (15)
N30—C31—C32	122.7 (3)	Cu1—O55—Cu2	103.52 (9)
C31—C32—H32	120.4	C58—O57—Cu1	114.07 (18)
C31—C32—C33	119.1 (3)	H60A—O60—H60B	119 (5)
C33—C32—H32	120.4	Cu1—O60—H60A	107 (3)
C32—C33—C34	119.1 (3)	Cu1—O60—H60B	122 (4)
O36—C33—C32	115.9 (3)	N10—Cu1—N20	80.60 (9)
O36—C33—C34	125.1 (3)	N10—Cu1—O60	97.51 (9)
C33—C34—H34	120.9	N20—Cu1—O60	89.96 (10)
C35—C34—C33	118.2 (3)	O55—Cu1—N10	161.28 (9)
C35—C34—H34	120.9	O55—Cu1—N20	99.09 (9)
C34—C35—C45	124.2 (2)	O55—Cu1—O57	85.65 (8)
N30—C35—C34	122.3 (2)	O55—Cu1—O60	101.20 (9)
N30—C35—C45	113.5 (2)	O57—Cu1—N10	91.69 (9)
H37A—C37—H37B	109.5	O57—Cu1—N20	168.93 (10)
H37A—C37—H37C	109.5	O57—Cu1—O60	98.98 (11)
H37B—C37—H37C	109.5	N30—Cu2—N40	81.11 (10)
O36—C37—H37A	109.5	N30—Cu2—O50	94.51 (10)
O36—C37—H37B	109.5	N30—Cu2—O55	111.51 (8)
O36—C37—H37C	109.5	N40—Cu2—O55	105.38 (8)
C42—C41—H41	119.1	O50—Cu2—N40	172.01 (10)
N40—C41—H41	119.1	O50—Cu2—O55	82.41 (8)
N40—C41—C42	121.9 (3)	O53—Cu2—N30	173.36 (10)
C41—C42—H42	120.2	O53—Cu2—N40	97.64 (9)
C41—C42—C43	119.5 (3)	O53—Cu2—O50	85.94 (9)
C43—C42—H42	120.2	O53—Cu2—O55	75.12 (7)
C42—C43—C44	119.0 (3)	H61A—O61—H61B	100 (4)
O46—C43—C42	116.1 (3)	H62A—O62—H62B	105 (4)
O46—C43—C44	124.9 (3)	H63A—O63—H63B	107 (4)
C43—C44—H44	121.1	H64A—O64—H64B	97 (4)
C45—C44—C43	117.9 (3)	H65A—O65—H65B	110 (4)
C45—C44—H44	121.1	H66A—O66—H66B	113 (4)
C44—C45—C35	123.9 (2)	H67A—O67—H67B	106 (4)
N40—C45—C35	113.3 (2)	H68A—O68—H68B	104 (4)
N40—C45—C44	122.8 (2)		
			
C11—C12—C13—C14	−0.6 (4)	C42—C43—C44—C45	0.7 (4)
C11—C12—C13—O16	179.8 (3)	C42—C43—O46—C47	−174.1 (3)
C12—C11—N10—C15	0.4 (4)	C43—C44—C45—C35	−179.0 (2)
C12—C11—N10—Cu1	−170.9 (2)	C43—C44—C45—N40	0.1 (4)
C12—C13—C14—C15	0.3 (4)	C44—C43—O46—C47	6.1 (4)
C12—C13—O16—C17	−179.2 (3)	C44—C45—N40—C41	−0.3 (4)
C13—C14—C15—C25	179.2 (2)	C44—C45—N40—Cu2	179.6 (2)
C13—C14—C15—N10	0.3 (4)	C45—C35—N30—C31	−178.6 (2)
C14—C13—O16—C17	1.2 (4)	C45—C35—N30—Cu2	5.5 (3)
C14—C15—C25—C24	4.0 (4)	C51—C54—C56—C58	−171.8 (2)
C14—C15—C25—N20	−175.8 (2)	C51—C54—C56—O55	66.2 (3)
C14—C15—N10—C11	−0.7 (4)	C51—C54—O53—Cu2	−41.2 (2)
C14—C15—N10—Cu1	171.4 (2)	C54—C51—O50—Cu2	3.5 (3)
C15—C25—N20—C21	−177.6 (2)	C54—C56—C58—O57	−118.9 (3)
C15—C25—N20—Cu1	2.6 (3)	C54—C56—C58—O59	61.3 (3)
C21—C22—C23—C24	2.7 (4)	C54—C56—O55—Cu1	119.29 (19)
C21—C22—C23—O26	−176.8 (3)	C54—C56—O55—Cu2	10.5 (2)
C22—C21—N20—C25	−2.7 (4)	C54—O53—Cu2—N40	−152.04 (16)
C22—C21—N20—Cu1	177.2 (2)	C54—O53—Cu2—O50	35.14 (17)
C22—C23—C24—C25	−2.7 (4)	C54—O53—Cu2—O55	−48.08 (15)
C22—C23—O26—C27	−179.3 (3)	C56—C54—O53—Cu2	75.3 (2)
C23—C24—C25—C15	−179.8 (2)	C56—C58—O57—Cu1	−2.3 (4)
C23—C24—C25—N20	0.0 (4)	C58—C56—O55—Cu1	−0.3 (3)
C24—C23—O26—C27	1.2 (4)	C58—C56—O55—Cu2	−109.1 (2)
C24—C25—N20—C21	2.6 (4)	N10—C11—C12—C13	0.2 (4)
C24—C25—N20—Cu1	−177.2 (2)	N10—C15—C25—C24	−177.0 (2)
C25—C15—N10—C11	−179.7 (2)	N10—C15—C25—N20	3.3 (3)
C25—C15—N10—Cu1	−7.7 (3)	N20—C21—C22—C23	0.0 (4)
C31—C32—C33—C34	0.2 (4)	N30—C31—C32—C33	0.5 (4)
C31—C32—C33—O36	−179.4 (3)	N30—C35—C45—C44	176.4 (2)
C32—C31—N30—C35	−0.8 (4)	N30—C35—C45—N40	−2.7 (3)
C32—C31—N30—Cu2	174.7 (2)	N40—C41—C42—C43	1.0 (4)
C32—C33—C34—C35	−0.5 (4)	O16—C13—C14—C15	180.0 (3)
C32—C33—O36—C37	−171.8 (3)	O26—C23—C24—C25	176.8 (3)
C33—C34—C35—C45	179.2 (2)	O36—C33—C34—C35	179.1 (3)
C33—C34—C35—N30	0.2 (4)	O46—C43—C44—C45	−179.5 (3)
C34—C33—O36—C37	8.5 (4)	O50—C51—C54—C56	−92.3 (3)
C34—C35—C45—C44	−2.7 (4)	O50—C51—C54—O53	26.1 (3)
C34—C35—C45—N40	178.2 (2)	O52—C51—C54—C56	84.0 (3)
C34—C35—N30—C31	0.5 (4)	O52—C51—C54—O53	−157.6 (3)
C34—C35—N30—Cu2	−175.4 (2)	O52—C51—O50—Cu2	−172.7 (2)
C35—C45—N40—C41	178.8 (2)	O53—C54—C56—C58	68.6 (3)
C35—C45—N40—Cu2	−1.2 (3)	O53—C54—C56—O55	−53.4 (3)
C41—C42—C43—C44	−1.2 (4)	O55—C56—C58—O57	1.8 (4)
C41—C42—C43—O46	179.0 (3)	O55—C56—C58—O59	−178.1 (3)
C42—C41—N40—C45	−0.2 (4)	O59—C58—O57—Cu1	177.5 (2)
C42—C41—N40—Cu2	179.8 (2)		

### Hydrogen-bond geometry (Å, º)

**Table:**

*D*—H···*A*	*D*—H	H···*A*	*D*···*A*	*D*—H···*A*
O60—H60*A*···O50^i^	0.81 (5)	2.00 (5)	2.802 (3)	167 (5)
O60—H60*B*···O67	0.77 (5)	1.98 (5)	2.750 (4)	177 (5)
O61—H61*A*···O65	0.81 (2)	2.02 (3)	2.813 (4)	166 (4)
O61—H61*B*···O52^ii^	0.82 (2)	1.92 (2)	2.739 (3)	177 (4)
O62—H62*A*···O64	0.79 (2)	2.01 (3)	2.774 (3)	164 (5)
O62—H62*B*···O55	0.80 (2)	1.85 (2)	2.650 (3)	174 (5)
O63—H63*A*···O59^ii^	0.83 (2)	1.97 (2)	2.806 (3)	178 (4)
O63—H63*B*···O61	0.85 (2)	2.13 (2)	2.970 (3)	176 (4)
O64—H64*A*···O52	0.81 (2)	2.05 (3)	2.762 (3)	146 (4)
O64—H64*B*···O65	0.85 (2)	1.95 (3)	2.719 (3)	149 (4)
O65—H65*A*···O59^iii^	0.82 (2)	2.08 (3)	2.871 (3)	162 (4)
O65—H65*B*···O66	0.81 (2)	2.01 (2)	2.801 (4)	167 (4)
O66—H66*A*···O53^ii^	0.79 (2)	1.91 (3)	2.680 (3)	169 (5)
O66—H66*B*···O62	0.80 (2)	2.02 (3)	2.808 (4)	167 (5)
O67—H67*A*···O62	0.84 (2)	1.91 (3)	2.737 (4)	168 (5)
O67—H67*B*···O59^ii^	0.83 (2)	2.15 (2)	2.973 (3)	178 (5)
O68—H68*A*···O57^iii^	0.90 (3)	2.52 (4)	3.236 (4)	138 (5)
O68—H68*B*···O60^iv^	0.91 (2)	2.23 (3)	3.129 (5)	169 (5)

Symmetry codes: (i) *x*+1, *y*, *z*; (ii) *x*, *y*, *z*−1; (iii) *x*−1, *y*, *z*−1; (iv) *x*−1, *y*, *z*.

## References

[bb1] Agens, M. C. (1960). US-American Patent No. 2938805.

[bb2] Allen, F. H. (2002). *Acta Cryst.* B**58**, 380–388. 10.1107/s0108768102003890 12037359

[bb4] Dolomanov, O. V., Bourhis, L. J., Gildea, R. J., Howard, J. A. K. & Puschmann, H. (2009). *J. Appl. Cryst.* **42**, 339–341.

[bb5] Farrugia, L. J. (2012). *J. Appl. Cryst.* **45**, 849–854.

[bb6] Fehling, H. (1848). *Arch. Physiol. Heilkd.* **7**, 64–73.

[bb7] Flack, H. D. & Bernardinelli, G. (2000). *J. Appl. Cryst.* **33**, 1143–1148.

[bb8] Groom, C. R., Bruno, I. J., Lightfoot, M. P. & Ward, S. C. (2016). *Acta Cryst.* B**72**, 171–179. 10.1107/S2052520616003954 PMC4822653 27048719

[bb9] Hesse, M., Meier, H. & Zeeh, B. (1979). *Spektroskopische Methoden in der organischen Chemie*, pp. 55–92. Suttgart: Thieme.

[bb10] Hörner, T. G. & Klüfers, P. (2016). *Eur. J. Inorg. Chem.* pp. 1798–1807.

[bb11] Jusys, Z. & Vaskelis, A. (1992). *Langmuir*, **8**, 1230–1231.

[bb12] Li, D.-S., Wang, Y.-Y., Liu, P., Luan, X.-J., Zhou, C.-H. & Shi, Q.-Z. (2005). *Acta Chim. Sinica*, **63**, 1633–1637.

[bb13] Li, D.-S., Zhou, C.-H., Wang, Y.-Y., Fu, F., Wu, Y.-P., Qi, G.-C. & Shi, Q.-Z. (2006). *Chin. J. Chem.* **24**, 1352–1358.

[bb14] Liu, J.-Q., Wang, Y.-Y., Ma, L.-F., Zhang, W.-H., Zeng, X.-R., Shi, Q.-Z. & Peng, S.-M. (2008). *Inorg. Chim. Acta*, **361**, 2327–2334.

[bb15] Macrae, C. F., Bruno, I. J., Chisholm, J. A., Edgington, P. R., McCabe, P., Pidcock, E., Rodriguez-Monge, L., Taylor, R., van de Streek, J. & Wood, P. A. (2008). *J. Appl. Cryst.* **41**, 466–470.

[bb16] Narcus, H. (1947). *Met. Finish.* **45**, 64–70.

[bb17] Oita, M., Matsuoka, M. & Iwakura, C. (1997). *Electrochim. Acta*, **42**, 1435–1440.

[bb18] Parsons, S., Flack, H. D. & Wagner, T. (2013). *Acta Cryst.* B**69**, 249–259. 10.1107/S2052519213010014 PMC3661305 23719469

[bb19] Rigaku OD (2015). *CrysAlis PRO*. Rigaku Oxford Diffraction, Yarnton, England.

[bb20] Saha, R., Biswas, S. & Mostafa, G. (2011). *CrystEngComm*, **13**, 1018–1028.

[bb21] Saubestre, E. B. (1972). *Plating* **59**, 563–566.

[bb22] Sheldrick, G. M. (2015*a*). *Acta Cryst.* A**71**, 3–8.

[bb23] Sheldrick, G. M. (2015*b*). *Acta Cryst.* C**71**, 3–8.

[bb24] Socrates, G. (2001). *Infrared and Raman Characteristic Group Frequencies*, 3rd ed. Chichester: Wiley.

[bb25] Van Den Meerakker, J. E. A. M. (1981). *J. Appl. Electrochem.* **11**, 387–393.

[bb26] Westrip, S. P. (2010). *J. Appl. Cryst.* **43**, 920–925.

[bb27] Wu, Y.-P., Fu, F., Li, D.-S., Yang, Z.-H., Zou, K. & Wang, Y.-Y. (2008). *Inorg. Chem. Commun.* **11**, 621–625.

